# Parathyromatosis: A Challenging Management in a Young Girl Case Report With Literature Review

**DOI:** 10.1155/crie/9239048

**Published:** 2025-10-24

**Authors:** Grace Jrad, Rim Masri, Bassam Abboud, Claude Ghorra, Charbel Mourad

**Affiliations:** ^1^Department of Endocrinology, Lebanese Hospital Geitaoui University Medical Center, Beirut, Lebanon; ^2^Faculty of Medical Sciences, Lebanese University, Hadat, Lebanon; ^3^Department of General Surgery, Lebanese Hospital Geitaoui University Medical Center, Beirut, Lebanon; ^4^Department of Anatomical Pathology, Lebanese Hospital Geitaoui University Medical Center, Beirut, Lebanon; ^5^Department of Diagnostic Imaging and Interventional Therapeutics, Lebanese Hospital Geitaoui University Medical Center, Beirut, Lebanon

**Keywords:** hypercalcemia, hyperparathyroidism, parathyroid adenoma, parathyromatosis

## Abstract

Parathytomatosis is a rare cause of persistent or recurrent hyperparathyroidism, defined by the presence of hyperfunctioning parathyroid tissues scattered throughout the neck and mediastinum. Preoperative diagnosis and localization of all the seeded parathyroid tissue is difficult, therefore, many imaging modalities are needed to determine the localization of these lesions for a more successful outcome. We report a case of a young girl with a severe primary hyperparathyroidism (PHPT) from a parathyroid adenoma, with renal and bone complications. She underwent parathyroidectomy and developed 2 years later recurrent disease in the form of parathyromatosis. We review the different tools for diagnosis and management since this condition remains a challenging issue. To our best knowledge, this case is the youngest case described in the literature and highlights the difficulties of management of parathyromatosis and the potential complications that may ensue from this disease.

## 1. Introduction

Primary hyperparathyroidism (PHPT) is a common endocrine disease caused by oversecretion of parathyroid hormone (PTH), most commonly from a single parathyroid adenoma. It affects 1% of the population, with a median age of 50–60 years [1] and a female predominance [2]. PHPT in adolescents is rare [1]. First line treatment consists of surgical removal which is the only curative treatment. If surgery fails to cure hyperparathyroidism, patients have either a persistent or recurrent disease [2, 3]. Persistent/recurrent PHPT is usually the result of failure to adequately remove the adenoma or all hyperplastic tissue [2, 4]. A rare cause of recurrence is parathyromatosis, which consists of benign hyperplasia of hyperfunctioning parathyroid tissue scattered throughout the thyroid bed region [2, 5, 6]. However, it can also result from hyperplasia of parathyroid rests from embryologic development. In both cases, it is considered the rarest yet challenging etiology of recurrent or persistent hyperparathyroidism [4, 5].

We describe here a rare case of PHPT in a 16-year-old girl complicated with a recurrent disease in the form of parathyromatosis presenting after first surgery for a parathyroid adenoma, with an associated ectopic parathyroid gland in the thymus. Our case is the youngest case of parathyromatosis described in the literature, reporting also the role of multimodality imaging in the preoperative localization of the different lesions.

## 2. Case Presentation

**Figure 1:**
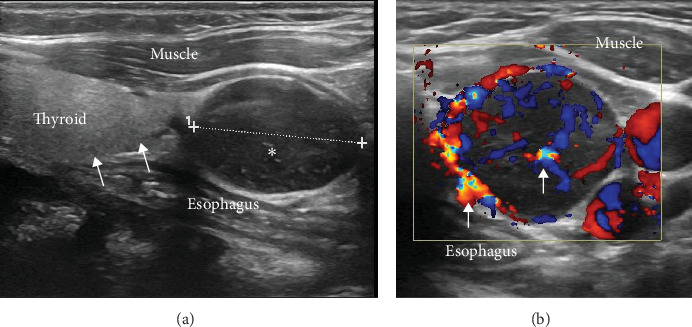
16-year-old girl with hyperparathyroidism. Longitudinal gray scale (A) and transverse color Doppler (B) ultrasound images of the left thyroid area. There is an oval well circumscribed 23 mm × 17 mm × 15 mm hypoechoic nodule (*⁣*^*∗*^ in subpart (A)) adjacent to the lower pole of the left thyroid lobe (arrows in subpart (A)). It shows increased central and peripheral vascularity on color Doppler (arrows in subpart (B)). Findings are consistent with a parathyroid adenoma.

A 16-year-old girl was referred to our outpatient clinic by her dentist after an incidental finding of a periodontal calcified granuloma. The patient did not complain of any clinical manifestations. Blood tests done showed a high serum calcium (Ca) of 14.2 mg/dL (8.4–10.8 mg/dL), with a low phosphorus level 2.3 mg/dL (3–4.5 mg/dL), a high serum PTH 944 pg/mL (9–75 pg/mL), and a creatinine level 0.9 mg/dL. Preoperative localization studies showed a unique left inferior parathyroid adenoma on ultrasound (Figure 1) hyperfixating on Tc-99 m sestamibi (MIBI) parathyroid scintigraphy.

**Figure 2:**
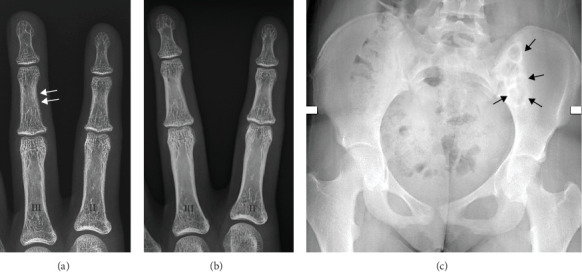
Bone changes before (A) and after treatment (B). Images were cropped and magnified from anteroposterior radiographs of the hands. II: index finger. III: third finger. (A) Before treatment, bone density is decreased, with a lace-like appearance of the cortex and subperiosteal bone resorption (arrows), signs of hyperparathyroidism. (B) After surgery and initiation of medical treatment, bone reconstruction with a normal appearance of bone density and cortex. (C) Anteroposterior radiograph of the pelvis showing a well circumscribed lytic lesion of the left iliac bone with sclerotic borders (arrows). In this context, it represents a brown tumor.

On further assessment, she was found to have skeletal manifestations of hyperparathyroidism (Figure 2) and early nephrocalcinosis on renal ultrasonography.

**Figure 3:**
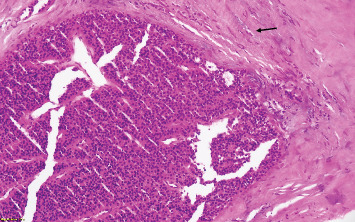
Histologic section from the initial surgical resection of the parathyroid adenoma, stained with hematoxylin and eosin (H&E), magnification ×20 showing a well-circumscribed nodule, composed predominantly of chief cells. The black arrow indicates a thick fibrous capsule.

The patient underwent a minimally invasive operation. The left-sided neck exploration revealed a grayish and enlarged left inferior parathyroid gland, located posterior to the lower pole of the thyroid. The gland was carefully dissected and excised without complications. Intraoperative frozen section analysis confirmed the parathyroid origin of the lesion, favoring an adenoma. Postoperative macroscopic and microscopic examinations confirmed the diagnosis of parathyroid adenoma showing a well-defined nodule weighing 2 g. The lesion was encapsulated, with a thick but clearly demarcated connective tissue capsule with no signs of malignancy (Figure 3).

The postoperative course was complicated by a hungry bone syndrome successfully treated in the hospital, and the patient was discharged on a maintenance dose of oral Ca and cholecalciferol.

**Figure 4:**
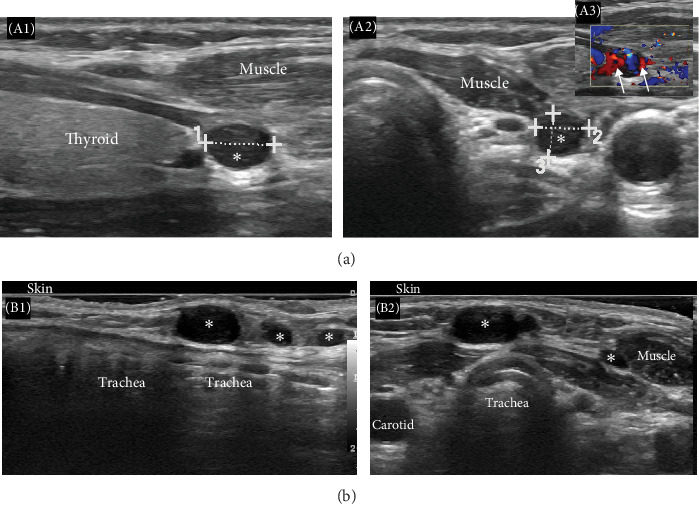
(A) Recurrent hyperparathyroidism 2 years after parathyroidectomy. Longitudinal (A1, B1) and transverse (A2, A3, and B2) grayscale ultrasound images of the left thyroid area. There is a 6.5 mm × 5 mm × 4.2 mm oval well circumscribed hypoechoic nodule (*⁣*^*∗*^ in subparts (A1, A2)) adjacent to the lower pole of the left thyroid lobe, at the site of previous parathyroidectomy. It shows increased vascularity on Doppler (arrows in subpart (A3)). Ultrasound characteristics are identical to the resected parathyroid adenoma and represent recurrent disease. (B) Recurrent hyperparathyroidism 2 years after parathyroidectomy. Longitudinal (B1) and transverse (B2) gray scale ultrasound images of the pretracheal area. There are multiple oval well circumscribed hypoechoic nodules (*⁣*^*∗*^ in subparts (B1, B2)) in the subcutaneous and muscular planes, the largest measures 12 mm. Ultrasound characteristics are identical to the resected parathyroid adenoma and represent parathyromatosis nodules.

After 2 years of uneventful follow-up, the patient consulted again for a palpable nonpainful pretracheal nodule along the surgical scar. Biological studies revealed a high Ca level (12.7 mg/dL), with hypophosphatemia (Ph 2.9 mg/dL) and an elevation of PTH of 185 pg/mL (9–75 pg/mL). Ultrasonography of the neck showed multiple hypoechoic well circumscribed nodules compatible with parathyroid adenomas in the subcutaneous and muscular planes (Figure 4a,b).

**Figure 5:**
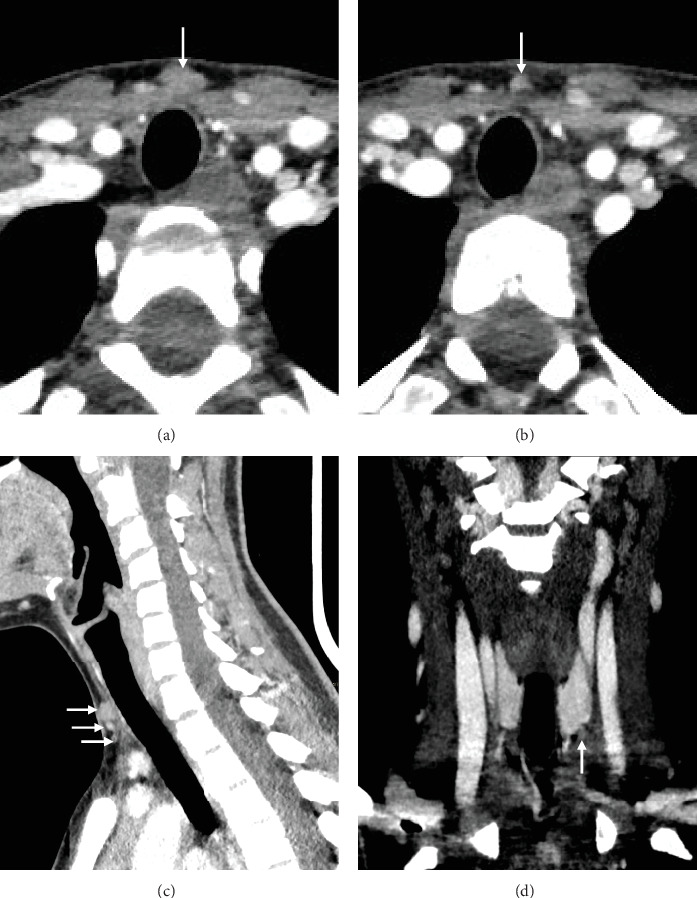
Recurrent hyperparathyroidism 2 years after parathyroidectomy. 4D computed tomography in the transverse (A, B), sagittal (C), and coronal (D) planes showing mildly enhancing nodules in the pretracheal subcutaneous tissue (arrows in subparts (A–C)) and at the lower pole of the left thyroid lobe (arrow in subpart (D)). Size ranges between 3 and 12 mm.

However, no uptake was detected on Tc-99 m sestamibi (MIBI) scintigraphy. These nodules were also confirmed on four dimensional computed tomography (4D CT) and no deep nodules were detected in the mediastinum (Figure 5). Multiple endocrine neoplasia (MEN) syndrome was ruled out by biological work up. The family did not consent for further genetic testing.

**Figure 6:**
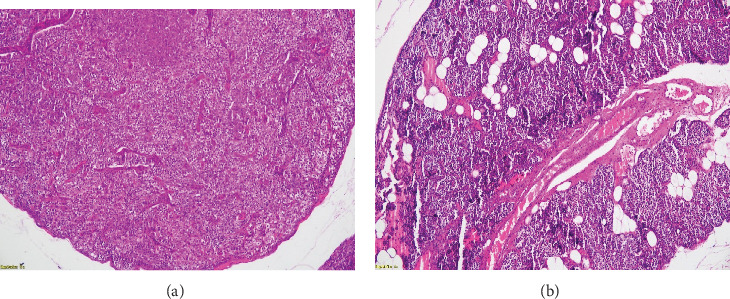
Histologic sections from the second surgical resection showing recurrent parathyroid nodules (A, B), stained with hematoxylin and eosin (H&E), magnification ×4. Multiple nodules of parathyroid tissue scattered within fibroadipose tissue consistent with parathyromatosis.

After a multidisciplinary discussion, the patient underwent an extensive surgical procedure with bilateral exploration by an expert surgeon, excising the parathyroid nodules previously described, the thymus, and the remaining parathyroid glands. The anatomopathological evaluation of the different lesions identified a 7 mm × 5 mm right subcutaneous nodule weighing 887 mg as an adenomatous parathyroid tissue. Similar histological features were observed in multiple coalescent right superior cervical nodules measuring 0.5 and 1.5 cm (Figure 6), confirming the presence of parathyroid tissue without signs of malignancy. Immunohistochemical staining for CDC73/parafibromin, which may aid in identifying hereditary forms of PHPT beyond MEN syndromes, could not be performed as it is not currently available at our institution. Our patient was diagnosed with parathyromatosis. Postoperatively, PTH and Ca levels were normal.

**Figure 7:**
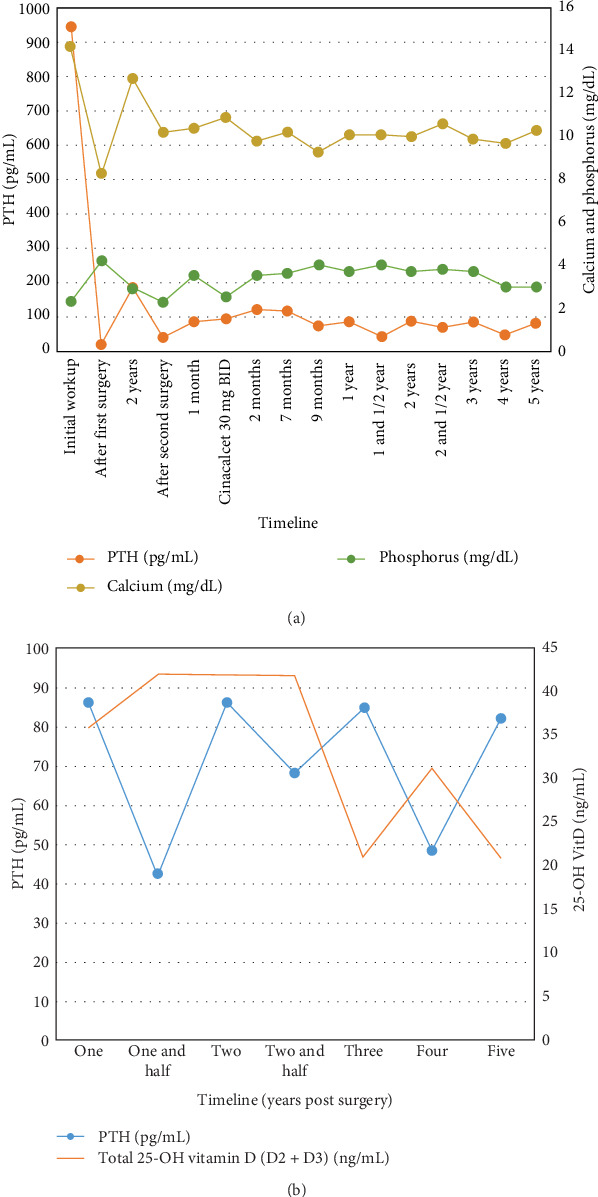
Time course during the management of the disease of (A) PTH level (left axis) and calcium and phosphorus (right axis) and (B) PTH level (left axis) and 25-OH vitamin D (right axis).

Two months later, PTH and Ca started to rise again. Parathyroid ultrasound showed two recurrent nodules on the left side. A decision to start calcimimetic was taken and the patient was put on Cinacalcet 30 mg oral twice daily, her serum Ca dropped to normal levels with a PTH level of 116 pg/mL (Figure 7A).

Bone densitometry revealed osteoporosis of the forearm (*Z*-score: −5.1) and left femur osteopenia.

Over the last 3 years, she has been followed up and kept in medical therapy with Cinacalcet 30 mg daily, with a current good control in serum Ca and phosphorus levels, ranging from 9.7 to 10.6 mg/dL and 3 to 4 mg/dL, respectively, and with normal renal function. However PTH level is changing insignificantly with minimal fluctuations of total 25-OH vitamin D2 + D3 (25-OH VitD) level (Figure 7B).

After 5 years, an ultrasound scan of the neck showed the presence of stable left-sided nodules. There is an improvement in the bone mineral density at the level of the distal radius (*Z*-score: −2.7) and the left femoral neck (*Z*-score: 0.5), while the density of the lumbar spine density (*Z*-score: −0.5) remained stable and the microarchitecture is normal (trabecular bone score measured 1.529 at L1–L4).

We presented a challenging case of parathyromatosis occurring in a young adult after an initial surgery for a single parathyroid adenoma, complicated with osteoporosis and nephrocalcinosis. Multiple imaging modalities were needed for an accurate preoperative diagnosis and follow-up.

## 3. Discussion

Parathyromatosis was first described by Palmer et al. [7] in 1975 in a series of 250 patients with multiple local recurrences after parathyroidectomy. It is an extremely uncommon cause of recurrent or persistent sporadic PHPT [8], and the incidence had not been recognized until this century. Only 64 cases have been reported in the medical literature [9], and just 28 reported cases in PubMed are related to PHPT [10].

Two types of parathyromatosis are identified. The primary form (Type 1) results from hyperplasia of parathyroid rests from embryologic development under the consistent physiological stimuli of PTH hormone seen most commonly in patients with end-stage renal disease (ESRD) with a frequency of 25% in renal failure [6, 11], but may also be a part of MEN syndromes [2, 4]. The secondary form (Type 2), also known as the most prevalent form of parathyromatosis, occurs after spillage and subsequent seeding of adenomatous or hyperplastic parathyroid tissue during parathyroidectomy or secondary implantation of cells into the surrounding soft tissue during percutaneous ablation from rupture of the capsule, typically in chronic kidney disease (CKD) [2, 4–6, 11]. Another case of parathyromatosis due to spontaneous rupture of a parathyroid adenoma was reported by Sim et al. [6] in 2013.

Renal osteodystrophy, soft tissue calcification, multiple fractures, muscle weakness, and bone/joint pain are complications related to parathyromatosis reported in a case series that are associated with a high mortality rate of up to 40% [12].

What we find peculiar in this case is the young age of onset (16 years) and the short disease-free interval, as well as the recurrence of secreting parathyromatosis nodules 2 years after resection of a benign parathyroid adenoma, without a known familial endocrine syndrome.

Our patient was 16 years old at the time of her first diagnosis of PHPT, with a recurrent parathyromatosis 2 years later, which distinguishes her from the cases reported in the literature. Although she had parathyroid tissue in her thymus, she did not have renal failure, MEN syndrome, or any other cause of parathyroid stimulation. Furthermore, the presence of a palpable lesion along the surgical scar and the presence of parathyroid tissue bilaterally and on the opposite side of the first surgery favor the hypothesis of a seeding of hyperactive parathyroid tissue during the primary operation. The relatively short time for recurrence of her hyperparathyroidism leads us to question whether our patient has an additional entity of low-grade malignancy with minimal local invasion [8], as described by Sim et al. [6]. This characteristic was also confirmed by many authors, with a mean time for reoperation ranging between 3 years in ESRD and 6 years in healthy subjects [2]. Others described a time period between 5 months and 19 years [11, 13]. If the time interval for the onset of the disease is shorter, a third theory of low-grade parathyroid carcinoma is to be discussed, especially when differentiating between parathyromatosis and parathyroid carcinoma is difficult to assess [4, 6, 11, 13–15].

Macroscopically, parathyromatosis appears as yellow–white tissue, and there is an overlap in the histologic features with atypical adenoma and parathyroid carcinoma. It mainly consists of oxyphilic cells microscopically, with no indication of necrosis, vascular invasion, nuclear pleomorphism, or increased mitotic activity. These features allow differentiation from parathyroid carcinoma [11, 15, 16]. Also, confirmation of a carcinoma requires the evidence of either clinical or histologic findings of local invasion of surrounding structures, the presence of a lymph node, or distant metastasis [6, 11, 15], which was not the case in our patient.

The preoperative diagnosis of parathyromatosis is usually challenging [17, 18]. To our best knowledge, there are no prospective studies comparing the diagnostic performance of the different imaging techniques in the diagnosis of parathyromatosis because it is a rare entity. Data in the literature consists of scattered case reports and small case series. In experienced hands, using high-end equipment, and in an adequate clinical context, ultrasound may have the highest sensitivity to detect superficial nodules in the neck. The use of color Doppler may be helpful to distinguish parathyromatosis nodules from scar tissue [5, 8, 11]. However, it is very limited in the evaluation of the mediastinum and the deeper areas of the neck. MIBI scintigraphy has a reduced sensitivity; this may be explained by the inherent lower spatial resolution of the technique, the small size of the nodules, which can also be nonfunctional [2]. One report showed that more nodules were detected using pinhole detectors when compared with parallel hole detectors [19]. 4D CT has a better performance to detect nodules in deeper locations (mediastinum, retrotracheal…). However, a considerable subset of parathyroid adenomas, and subsequently parathyromatosis nodules, do not have the typical enhancement characteristics on 4D CT and cannot be easily distinguished from lymph nodes or scar tissue [20–23]. The role of PET-Choline is yet to be defined. The combination of imaging techniques is, therefore, advocated for a thorough preoperative localization and multidisciplinary management.

The management of parathyromatosis is associated with a high failure rate with both surgery and medical treatment. The first line of treatment remains an extensive surgery, including resection en-bloc of all the macroscopic foci of parathyroid lesions, but also thyroidectomy, thymectomy (high rate of ectopic parathyroid glands), and clearance of both the central neck compartment and mediastinum [2, 6, 8, 11]. However, the biggest challenge in these surgeries is to identify all the disseminated tiny nodules, especially those adherent to scar tissue or surrounding structures [2].

Therefore, surgery does not often lead to a complete cure especially with difficulties regarding localizing and identifying all the nodules preoperatively. Medical therapy is then needed to control hypercalcemia and to achieve a normocalcemic state [6].

Cinacalcet, an oral calcimimetic, is currently approved for use in secondary hyperparathyroidism in patients with CKD on dialysis, in parathyroid carcinoma with hypercalcemia, and in patients with PHPT for whom parathyroidectomy is not feasible. It has been the most successful medical therapy to date for parathyromatosis. Its dose ranges from 60 to 180 mg/day for 3–26 months [5, 6, 11, 24]. In our patient, Cinacalcet was administered over 3 years, initially at a dose of 60 mg per day, then reduced to 30 mg daily for the last 5 years. The tolerability was excellent, and the effectiveness in controlling serum Ca levels was sustained.

This drug increases the sensitivity of the Ca sensing receptor on the parathyroid gland, lowering PTH levels, serum Ca, and phosphorus. It was also demonstrated that it can protect against bone loss [2, 5]. In addition, it can inhibit proliferation of parathyroid cells and may increase apoptosis of parathyroid cells at high doses [5, 6].

Bisphosphonates, especially alendronate, are also used and are found to be efficient in patients with parathyromatosis and associated osteoporosis, with stabilization of bone mineral density. However, there is only subtle and transient effects on Ca levels [2, 6, 11]. Vitamin D supplements lead to a reduction in PTH levels and a decrease in bone turnover [11].

There have also been reports on long-term usage of denosumab as therapy, which is a humanized monoclonal antibody. Its inhibition of the receptor activator of the nuclear factor κ-B ligand (RANKL)/RANK signaling pathway can be effective in blunting the catabolic effect of PTH in the bone [2, 5, 7, 11, 25]. Reports on the use of somatostatin analogs, such as Lanreotide, for long-term therapy have also emerged in the treatment of PHPT, especially in patients with MEN1 syndrome, but with conflicting results [9].

Traditional medical therapy and surgical approaches appear to be of limited value since in many studies, multiple surgeries were required with difficulties in controlling serum Ca levels and achieving complete remission [8]. Therefore parathyromatosis can be associated with serious complications related to the chronic hypercalcemia, mainly affecting bone density.

Although our patient has responded well to calcimimetic therapy thus far, the potential for long-term disease control is unknown. Bisphosphonates were not considered in our case due to lack of evidence on their use in young osteoporotic patients and the presence of one study showing an increase of bone density of 11% at the forearm after 1.4 years of treatment with Cinacalcet [2].

## 4. Conclusion

To the authors' best knowledge, our patient is among the youngest cases of parathyromatosis described in the literature, presenting 2 years after her parathyroidectomy for a benign adenoma. We emphasize the role of a multidisciplinary management, together with a combination of multiple imaging modalities for this rare challenging diagnosis. Unfortunately, a genetic cause cannot be entirely excluded and the diagnosis of parathyromatosis Type 2 remains presumptive.

## Data Availability

All data generated or analyzed during this study are included in this article.

## Nomenclature

PHPT: Primary hyperparathyroididsm
PTH: Parathyroid hormone
MIBI: Tc-99 m sestamibi
Ca: Calcium
Ph: Phosphorus
MEN syndrome: Multiple endocrine neoplasia syndrome
25-OH VitD: Total 25-OH vitamin D2 + D3
ESRD: End-stage renal disease
CKD: Chronic kidney disease
4D CT: Four dimensional computed tomography
RANK: Receptor activator of the nuclear factor κ-B
RANKL: Receptor activator of the nuclear factor κ-B ligand.
